# Clozapine-induced inflammation: a case report

**DOI:** 10.3389/fpsyt.2026.1886320

**Published:** 2026-08-28

**Authors:** Tian Han, Iunan Lin, Xiao Ji

**Affiliations:** Beijing Key Laboratory of Intelligent Drug Research and Development for Mental Disorders & National Clinical Research Center for Mental Disorders & National Center for Mental Disorders & Beijing Anding Hospital, Capital Medical University, Beijing, China

**Keywords:** Clozapine, C-reactive protein, inflammatory response, treatment-resistant mania, white blood cells

## Abstract

**Introduction:**

Clozapine is highly effective for treatment-resistant schizophrenia, schizoaffective disorder, and treatment-resistant mania. However, it is associated with significant inflammatory adverse effects, including fever and severe organ involvement such as myocarditis, pneumonia, and enteritis. Close serial monitoring of inflammatory markers (white blood cell count, C-reactive protein, and neutrophils) combined with individualized, slow dose titration is essential to minimize these risks.

**Case report:**

We describe a 21-year-old Chinese male with treatment-resistant mania who developed fever, pulmonary inflammation, myalgia, nausea, vomiting, abdominal distension, leukocytosis with neutrophilia, and elevated C-reactive protein during the initial rapid titration of clozapine. The inflammatory response resolved promptly after dose reduction and did not recur with a slower titration schedule and lower maintenance dose.

**Conclusions:**

This case highlights the importance of vigilant monitoring of inflammatory markers during clozapine initiation and supports the use of personalized titration regimens, particularly in East Asian populations who may be at heightened risk of clozapine-induced inflammation.

## 1 Introduction

Clozapine is an atypical antipsychotic with well-established efficacy in reducing suicide risk and improving outcomes in treatment-resistant schizophrenia. Evidence also supports its use in refractory mania and bipolar disorder among patients who respond poorly to or cannot tolerate conventional mood stabilizers and other antipsychotics. Multiple studies have demonstrated clozapine’s effectiveness in managing refractory manic episodes—particularly in bipolar I disorder—by controlling agitation, aggression, psychotic symptoms, and mood instability (1). International guidelines often position clozapine as a third-line or last-resort option for refractory mania, typically after failure of adequate trials of at least two mood stabilizers (e.g., lithium and valproate) and two second-generation antipsychotics (e.g., olanzapine and quetiapine) (2). For patients with prominent psychotic features, clozapine may offer superior antipsychotic effects compared with traditional mood stabilizers (3).

Nevertheless, clozapine’s use is limited by potentially serious inflammatory adverse reactions, including myocarditis, pneumonia, and drug reaction with eosinophilia and systemic symptoms (DRESS) syndrome. Recent literature emphasizes the value of early detection of clozapine-induced inflammation, which can herald more severe organ damage. This report presents a case of treatment-resistant mania in which the patient developed an inflammatory response during rapid clozapine titration. We discuss the clinical course, differential diagnosis, management, and implications for individualized pharmacotherapy.

## 2 Case presentation

A 21-year-old male presented with an 8-year history of mood instability characterized by alternating periods of low mood and excitement, accompanied by disorganized speech. In the 6 months preceding admission, he exhibited prominent excitement and disorganized speech.

The patient was first hospitalized in 2015 for depressive symptoms with suicidal ideation and auditory hallucinations. He was diagnosed with a depressive episode with psychotic features and treated with fluvoxamine, magnesium valproate, and aripiprazole, with good response. He discontinued medications after studying abroad in 2022.

In January 2023, he developed emotional lability, irritability, hyperactivity, decreased sleep, auditory hallucinations, pressured speech, and disorganized thinking. He was diagnosed with bipolar disorder, manic episode with psychotic features, and treated with lithium carbonate (2.25 g/day), olanzapine (30 mg/day), and sodium valproate (0.4 g/day) without improvement.

After returning to China in March 2023, he underwent two hospitalizations and received 24 sessions of modified electroconvulsive therapy (MECT). Pharmacotherapy included sodium valproate sustained-release (1.5 g/day), lithium carbonate (1.25 g/day), and quetiapine (0.7 g/day), later switched to aripiprazole (30 mg/day) due to paroxysmal supraventricular tachycardia. Risperidone (up to 7 mg/day) was added but yielded no significant benefit.

Due to the patient’s extreme agitation, the previous psychiatrist decided to switch from risperidone to clozapine and attempted to achieve rapid sedation by quickly titrating the clozapine dose. However, the psychiatrist did not follow guideline recommendations regarding the appropriate rate of clozapine dose titration or the monitoring required during the titration period (such as monitoring the complete blood count, drug levels, and C-reactive protein). On July 3, 2023, risperidone was reduced to 4 mg/day, and clozapine was started at 75 mg/day, then gradually increased to 275 mg/day over 7 days. Afterward, maintenance treatment was continued with sodium valproate (1.0 g/day) and lithium carbonate (1.25 g/day). Following these adjustments, the patient’s symptoms did not improve, and adverse effects including muscle aches, nausea, vomiting, and abdominal distension developed, prompting transfer to the emergency department of a psychiatric specialty hospital.

The patient, diagnosed with paroxysmal supraventricular tachycardia in 2023, presented with a BMI of 28.6 and tachycardia (120 bpm). Mental status exam showed pressured speech, flight of ideas, agitation, auditory hallucinations, and poor insight, meeting ICD-11 criteria for bipolar disorder with a current manic episode with psychotic symptoms. This episode lasted over 6 months and was treatment-resistant, failing two mood stabilizers, four antipsychotics, and MECT.

On the second day after admission — which was also the 7th day of clozapine treatment (at a dose of clozapine 275 mg/day) — the patient developed fever (peak 38.6 °C) accompanied by sialorrhea, pharyngeal discomfort, cough, palpitations, and bilateral lower limb myalgia. Chest CT revealed multifocal pneumonia. Laboratory findings showed elevated creatine kinase, C-reactive protein (CRP), white blood cell count, and neutrophils. He was diagnosed with pneumonia and started on cefoperazone–sulbactam. After clozapine dose reduction (at a dose of clozapine 225 mg/day) and 3 days of antibiotics, fever and blood counts normalized, with near-complete resolution of pneumonia on repeat CT.

On the 11th day of clozapine treatment, the patient developed a recurrent fever (39.2 °C) accompanied by chills, cough, and productive sputum. Inflammatory markers rose again. By day 17, although the fever was ongoing, the patient remained in a state of extreme agitation and irritability. Antibiotic therapy was switched to sitafloxacin, and an extensive infectious workup—including viral serologies, blood cultures, procalcitonin, and testing for Brucella—returned negative. The fever was therefore attributed to clozapine. To avoid noncompliance with diagnostic procedures for the fever workup, the treatment team consulted with a pulmonologist. It was decided not to discontinue clozapine completely but rather to reduce the dose to 50 mg/day. At the same time, doses of valproate and lithium were increased to maintain mood stability. Following this adjustment, the fever resolved completely. However, 13 days later, mood symptoms recurred, prompting the patient to be transferred for further care.

Since both lithium and valproate had already reached their maximum therapeutic concentrations, yet mood symptoms persisted, and the patient’s inflammatory response had resolved after reducing clozapine to 50 mg/day, the psychiatric team decided to slowly reintroduce and titrate clozapine in order to better control the patient’s manic symptoms. Under pharmacist guidance, clozapine was slowly titrated—starting at 75 mg/day and increased to 100 mg/day after 7 days. Therapeutic levels of valproate and lithium were maintained throughout, and no further inflammatory reactions occurred.

**Figure 1:**
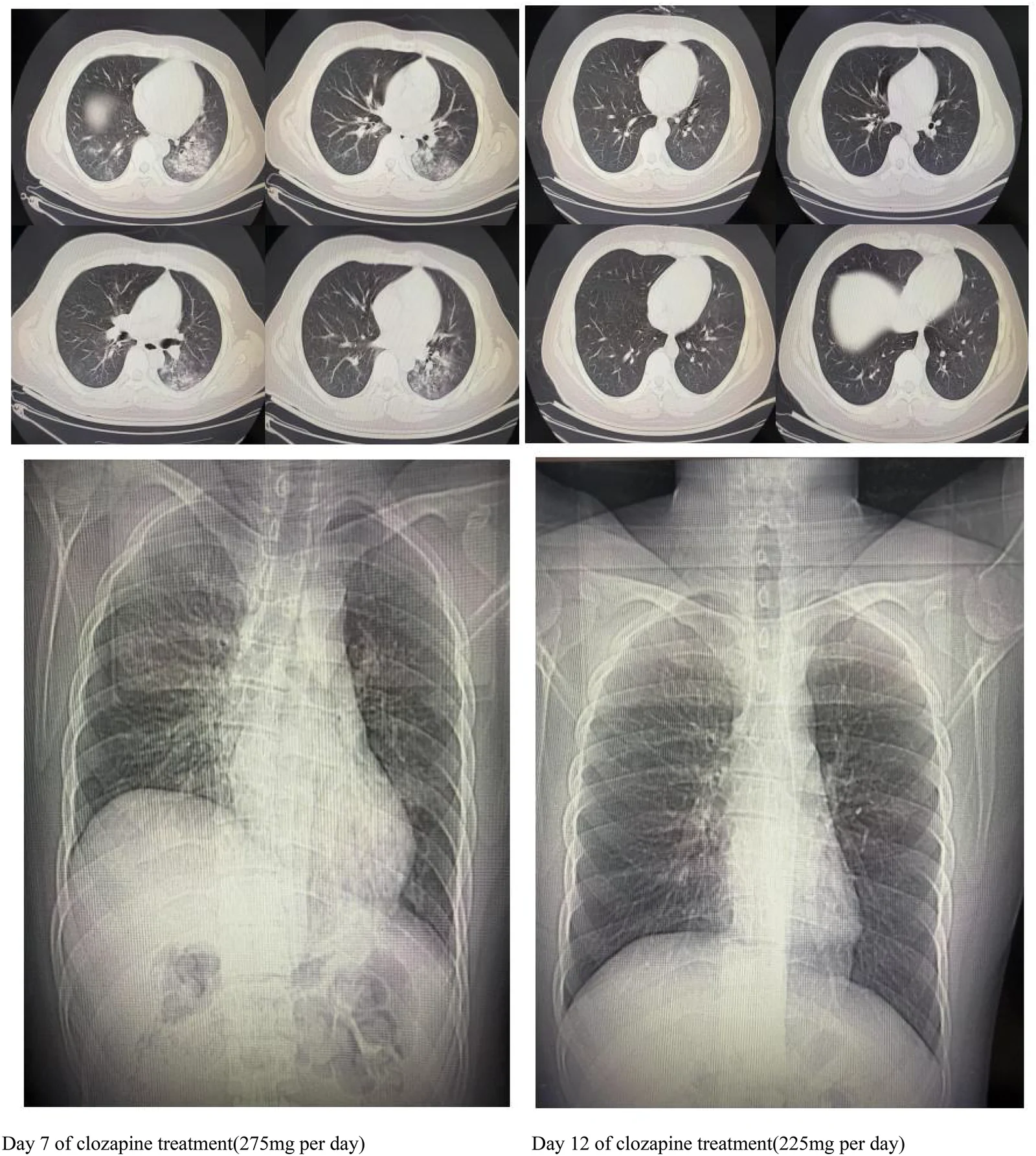
Chest CT.

**Figure 2:**
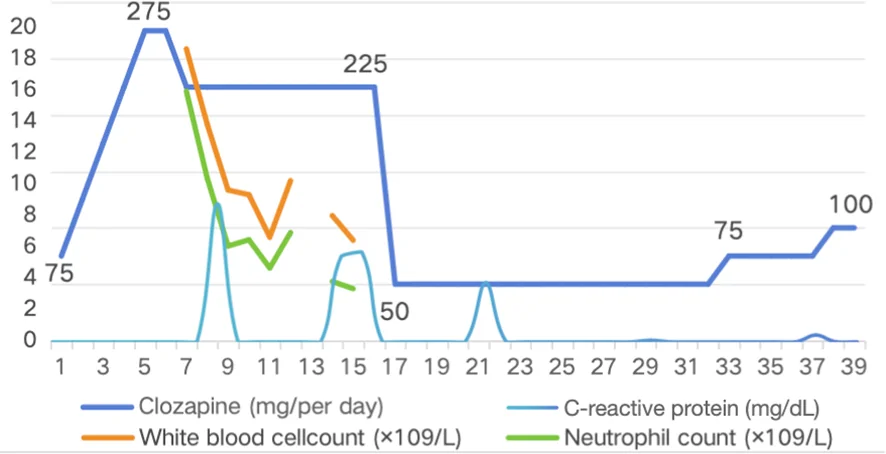
The relationship between the dose adjustment of clozapine before treatment and white blood cell count, neutrophil count, C-reactive protein.

Medication adjustments, body temperature (**Table 1**), inflammatory markers and drug levels (**Table 2**), chest CT images (**Figure 1**), and the relationship between clozapine dose and inflammatory markers (**Figure 2**) are presented below.

**Table 1:** Drug dosage and body temperature.

Time to start clozapine treatment	Body temperature (°C)	Clozapine (mg/per day)	Valproate (g/per day)	Lithium (g/per day)	Risperidone (mg/per day)
1	-	75	1.0	1.25	7
3	-	175	1.0	1.25	4
6	36.8	275	1.25	1.25	2
7	38.5	225	1.25	1.25	-
8	36.8	225	1.5	1.25	–
9	36.8	225	1.75	1.25	-
10	36.8	225	1.75	1.25	–
11	39.2	225	1.75	1.25	-
12	38.9	225	1.75	1.25	–
17	36.8	50	2.0	1.25	-
33	36.7	75	2.0	1.0	–
38 days later	36.8	100	1.75	1.25	-

**Table 2:** Inflammatory markers and drug concentrations.

Time to start clozapine treatment	White blood cell count (10^9^/L)	Neutrophil count v(10^9^/L)	C-reactive protein (mg/dL)	Clozapine concentration(ng/ml)	Valproic acid concentration(μg/ml)	Lithium concentration(mmol/L)	Risperidone concentration (ng/ml)
3	10.7 ↑	7.06 ↑	-	-	76.17	0.65	40.13
7	18.6 ↑	15.9 ↑	–	328.96	77.39	0.71	24.39
8	13.8 ↑	10.36 ↑	8.43 ↑	-	-	-	-
9	9.6 ↑	6.03	–	–	–	–	–
10	9.3	6.43 ↑	-	238.09	99.62	0.99	-
11	6.6	4.63	–	–	–	0.71	–
12	10.2 ↑	6.89 ↑	-	-	-	-	-
14	7.97	3.77	5.23 ↑	477.23	83.66	1.18	
15	6.40	3.31	5.47 ↑				
21	10.2 ↑	6.55 ↑	3.61 ↑	136.12	86.07	0.88	–
29	13.56 ↑	8.22 ↑	0.1				
34	–	–	–	91.57	91.41	0.77	–
37	8.3	7.51 ↑	0.42	80.10	110.47	0.92	-
39	–	–	–	–	94.96	–	–
42	10.4 ↑	4.8	-	141.97	83.54	0.71	-
44	9.8 ↑	4.76	–	–	–	–	–
47	-	-	-	-		0.93	-
54	7.7	51.3	0.28	104.10	84.15	0.71	

This arrow means the amount is higher than normality.

The patient was followed for 2 years post-discharge on clozapine 100 mg/day, sodium valproate sustained-release 1.5 g/day, and lithium carbonate 1.0 g/day. He remained in sustained remission.

## 3 Discussion

This case illustrates clozapine-induced systemic inflammation in a young Chinese male with treatment-resistant mania during rapid dose titration. The presentation included fever, leukocytosis with neutrophilia, elevated CRP, and pulmonary infiltrates—features consistent with clozapine-associated inflammatory responses, which are most common early in treatment (4). Prospective data from Japanese patients have shown dynamic increases in inflammatory cytokines (e.g., IL-6) during titration (5). Similar inflammatory phenomena have been reported with Sweet syndrome (6), acute interstitial nephritis (7), and drug-induced pneumonitis (8).

Research suggests that the core mechanism underlying clozapine-induced inflammatory reactions involves abnormal activation of the immune system. Both *in vitro* and animal model studies have shown that clozapine can activate inflammasomes in monocytes, leading to caspase-1 activation and the release of pro-inflammatory cytokines such as IL-1β (9). Clozapine may also elevate levels of leptin and pro-inflammatory cytokines, thereby influencing immune function (10). The inflammatory response itself can suppress the activity of cytochrome P450 1A2 (CYP1A2)—the primary enzyme responsible for clozapine metabolism in the liver—creating a vicious cycle: inflammation raises clozapine plasma concentrations, and higher concentrations may in turn exacerbate inflammation (10–12). Inhibition of CYP1A2 can lead to drug accumulation, increasing the risk of pneumonia (10). In the early phase of clozapine treatment, rapid dose titration has also been associated with neutropenia, which may increase infection risk (13). During the peak of inflammation, the required dose correction factor may be as low as 0.4 to 0.69 times the usual dose—meaning the dose may need to be reduced by nearly half to one-third (12). Therefore, whenever infection or inflammation occurs, clinicians should consider halving the clozapine dose to prevent drug toxicity (13).

East Asian populations, including Chinese individuals, exhibit reduced clozapine clearance due to CYP1A2 polymorphisms and lifestyle factors (e.g., nonsmoking status, higher BMI), leading to higher plasma concentrations and increased risk of adverse effects (10). This patient had multiple risk factors: East Asian ancestry, nonsmoking, obesity (BMI 28.6 kg/m²), and concomitant valproate (a CYP inhibitor) (14). Clozapine levels peaked at approximately 407 ng/mL during the initial rapid titration.

The case exemplifies the “continuum of severity” of clozapine-induced inflammation, progressing from subclinical marker elevation to overt organ involvement (15, 16). Negative infectious workup and lack of response to antibiotics strongly supported a drug-related etiology.

Importantly, the initial rapid titration (75 mg to 275 mg in ~5 days) deviated from emerging ancestry-based recommendations for slower titration in East Asians (≤25 mg/week increments with weekly CRP monitoring for the first 4 weeks) (15, 17). The subsequent slow retitration to a modest maintenance dose (100 mg/day), combined with optimized mood stabilizers, achieved sustained remission without recurrence of inflammation. This underscores the value of personalized, safety-focused strategies. Concomitant valproate further necessitates cautious dosing (18).

Fever during clozapine titration prompted consideration of neuroleptic malignant syndrome (NMS); however, the absence of altered mental status, rigidity, or markedly elevated creatine kinase ruled out NMS.

## 4 Conclusions

Clozapine remains an important therapeutic option for treatment-resistant mania, but early inflammatory reactions represent a potentially serious yet often underrecognized adverse effect, particularly in Chinese and other East Asian patients who may have reduced clozapine clearance. This case demonstrates that rapid dose escalation, together with predisposing factors such as East Asian ancestry, obesity, nonsmoking status, and concomitant valproate therapy, may precipitate systemic inflammation and secondary elevation of clozapine exposure.

Importantly, our case also shows that clozapine-induced inflammation does not necessarily require permanent discontinuation of clozapine. After exclusion of alternative causes and careful clinical assessment, marked dose reduction followed by individualized slow retitration under close laboratory monitoring successfully controlled the inflammatory response while preserving the long-term therapeutic benefits of clozapine. This strategy resulted in sustained psychiatric remission without recurrence of inflammation during two years of follow-up.

Based on this experience and the emerging evidence supporting ancestry-based dosing strategies, we recommend:

Personalized titration: Initiate clozapine at 12.5–25 mg/day and increase slowly (preferably no more than 25 mg/week in East Asian patients). If inflammatory reactions occur and immediate discontinuation is not clinically feasible, reduce the dose to the starting dose or lower (e.g., 12.5–50 mg/day), monitor CRP, neutrophil-to-lymphocyte ratio, and clozapine plasma concentrations, and resume titration only after inflammatory markers normalize.

Intensive monitoring: Perform weekly CRP and complete blood count monitoring during at least the first four weeks of treatment, with more frequent assessments in high-risk patients or when fever develops.

Risk factor assessment: Exercise additional caution in patients with reduced CYP1A2 activity, including East Asian individuals, nonsmokers, obese patients, those receiving concomitant valproate, and older adults, as these factors may increase clozapine exposure and susceptibility to inflammation.

Greater awareness of clozapine-induced inflammation, combined with individualized dosing and proactive monitoring, may improve the safe use of clozapine while avoiding unnecessary treatment discontinuation. Future prospective studies are needed to validate ancestry-based titration protocols and determine the optimal biomarker-guided strategies for preventing inflammatory complications during clozapine initiation.

## Data Availability

The raw data supporting the conclusions of this article will be made available by the authors, without undue reservation.
